# London Calls? Discrimination of European Job Seekers in the Aftermath of the Brexit Referendum

**DOI:** 10.3389/fsoc.2021.737857

**Published:** 2021-12-22

**Authors:** Valentina Di Stasio, Anthony Francis Heath

**Affiliations:** ^1^ Utrecht University, Utrecht, Netherlands; ^2^ European Research Centre on Migration and Ethnic Relations (ERCOMER), Utrecht, Netherlands; ^3^ Centre for Social Investigation, Nuffield College, Oxford University, Oxford, United Kingdom

**Keywords:** discrimination, employers, field experiment, Europeans, Brexit, public opinion, English regions, London

## Abstract

The central question in this article is whether there was greater discrimination against European applicants in the labor market in those English regions where public opinion was more strongly in favor of Brexit. Using a field experiment conducted immediately after the Brexit Referendum, we provide causal evidence that applicants with EU backgrounds faced discrimination when applying for jobs in England. On average, applicants from EU12 countries and applicants from Eastern European member states were both less likely to receive a callback from employers than were white British applicants. Furthermore, in British regions where support for Brexit was stronger, employers were more likely to discriminate against EU12 applicants. This finding, though, is driven by the more favorable treatment reserved to EU12 applicants applying for jobs in the Greater London area. Eastern Europeans, on the other hand, did not benefit from this ‘London advantage’. Administrative and legal uncertainties over the settlement status of EU nationals cannot explain these findings, as European applicants, both EU12 and Eastern Europeans, faced the same legislative framework in all British regions, including London. Rather, London appears to exhibit a cultural milieu of ‘selective cosmopolitanism’. These findings add to the still limited literature on the relationship between public opinion on immigrants (here proxied by the referendum vote) and the levels of ethnic discrimination recorded in field experiments.

## Introduction

On June 23, 2016, more than 17 million voters cast their preference for the United Kingdom to leave the European Union (EU). The “Brexit” referendum was won by the Leave campaign by a slim margin—51.9% voted for Leave *vs*. 48.1% for Remain—and was followed by economic turbulence and a political stalemate (Electoral Commission, 2016). Immigration of EU nationals to the United Kingdom and the desire to take back control over immigration were key issues in the public debate leading to the referendum, which was criticized for being “an over simplified and highly emotional in-out choice” (O’Reilly 2016: 811). Brexit has been widely interpreted as an example of the populist nationalism that has been resurgent in Western democracies, and EU nationals living in Britain perceived that their own right to reside in the country was at stake and interpreted the referendum result as “a vote on immigrants” (Lulle et al., 2018: 6).

Alarmingly, a sharp rise in racially or religiously aggravated hate crimes was observed in Britain around the time of the referendum (O’Neill, 2017). Next to evidence from police records, qualitative studies of EU nationals, especially Poles, pointed to episodes of bullying, harassment, verbal abuse and name-calling in several life domains, including access to services, employment, relations at school and with neighbors (e.g., Benedi Lauherta and Iusmen, 2019; Rzepnikowska, 2018). The general climate of hostility and uncertainty made EU nationals feel unwelcome, vulnerable and powerless, and some even reconsidered their intention to stay (Lulle et al., 2018; Ranta and Nancheva, 2019). These feelings were not only shared by Europeans who moved to Britain as adults for work-related reasons. Young people too, that is the 1.5 migrant generation, perceived Brexit as a rupture in their developing sense of belonging to Britain (Tyrrell et al., 2019).

While the British government was negotiating the terms of the withdrawal agreement, a crucial issue was how to formally regulate the residence status of more than three million European nationals living and working in the United Kingdom. Theresa May, Prime Minister at the time, pledged that EU nationals lawfully residing in the country would be granted the right to stay and offered an easy route to settlement. However, administrative and legal uncertainty remained and EU nationals trying to gain long-term residence rights encountered a generally hostile environment when dealing with the United Kingdom Immigration Service (Benedi Lauherta and Iusmen, 2019). Growing evidence, collected by the Labour party and the3million (a grassroots organization campaigning for EU citizens’ rights) as well as news media, revealed that landlords and employers were unlawfully restricting tenancies or job openings to British passport holders or asking EU nationals to provide copies of their settled status documentation (e.g., The Guardian, 2017; SkyNews, 2019). Moreover, the share of EU-born respondents living in Britain who identified in the European Social Survey as members of a group facing discrimination on grounds of color, race, nationality, religion, language or ethnicity doubled between the years 2010–12 and 2014–16 (Fernandez-Reino, 2020).

In this study, we examine whether applicants with EU backgrounds faced a similarly hostile environment when applying for jobs. We study discrimination in hiring decisions, drawing on a field experiment we conducted in Britain in the immediate aftermath of the Brexit referendum. The fieldwork took place between August 2016 and December 2017. We randomly assigned either British-sounding or foreign-sounding names to fictitious job applications, an experimental design which allows us to compare the responses (callbacks) received by white British applicants to those received by applicants of European background. As the applications were identical in terms of skills, qualifications and job-related characteristics, we interpret differences in callbacks as evidence of discrimination, a state-of-the-art approach in the literature (for reviews, see Blank et al., 2004; Heath and Di Stasio, 2019; Pager, 2007).

Our design includes applicants originating from some of the most popular sending countries in the EU-born United Kingdom population (Vargas-Silva and Fernandez-Reino, 2018). In a more nuanced analysis, we can then test whether discrimination only affects applicants from Eastern European countries that joined the EU after 2004 (Bulgaria, Poland, Romania), or also EU12 applicants, originating from France, Germany Ireland, Netherlands, Greece, Italy, Spain. We also examine whether the callback gap between white British and EU applicants widens in regions characterized by a higher share of votes for Brexit.

Our contribution to the literature is threefold. First, we add to an emerging line of research on the impact of Brexit on the subjective and objective vulnerability experienced by EU nationals in the aftermath of the referendum, and in particular on its human resourcing implications (Ridgway 2019). With our field experiment, we provide causal evidence that EU applicants faced discrimination when applying for jobs in England. On average, applicants from EU12 countries and applicants from Eastern European member states were both less likely to receive a callback than were white British applicants. At the same time, the disadvantage they faced is relatively modest, especially if compared with the treatment afforded to non-white ethnic minorities, and concentrated in non-graduate occupations such as cooks, admin and clerk jobs. Second, we broaden the geographical reach of field experiments on hiring discrimination that, with a few exceptions (Koopmans et al., 2019; Thijssen et al., 2019), have so far limited their focus to non-Western ethnic minorities or compared the latter to a single European group (Baert et al., 2017; McGinnity and Lunn, 2011). Third, our analysis reveals that only EU12 applicants benefitted from the cosmopolitanism of the Greater London Area, where they were treated on a par with the white British group. In the other British regions, where support for Brexit was stronger, employers were more likely to discriminate against EU12 applicants. Administrative and legal uncertainties over the settlement status of EU nationals cannot convincingly explain the lack of a “London advantage” for Eastern Europeans, as all EU nationals were subject to the same legislative framework. An alternative interpretation is that London provides a distinctive cosmopolitan context (albeit a selective one) in which stereotyped thinking is less embedded. These findings add to the still limited literature on the relationship between public opinion on immigration, here proxied by the referendum vote, and the levels of discrimination against migrants recorded in field experiments (Carlsson and Rooth 2012; Carlsson and Eriksson, 2017).

## Theoretical Framework

### Euroscepticism and the Europeanization of Immigration to the United Kingdom

In the aftermath of the referendum, a growing body of research on populism and Eurosceptic voting has examined the drivers of the Brexit vote. The proposed explanations, which we summarize below, fit neatly into the distinction between utilitarian (instrumental) and identity approaches to the study of public opinion on European integration (Hobolt and De Vries, 2016; Hooghe and Marks, 2005). First, at the macro-level, structural explanations pointed to the geographical concentration of economic distress in low-productivity regions: areas with high unemployment, limited real wage growth, a sharp decline in manufacturing and long-term economic decline were systematically related to the Leave vote (Becker et al., 2017; Colantone and Stanig, 2018; Dijkstra et al., 2019; Blackaby et al., 2020). Second, at the micro-level, individual-level explanations focused on the socio-economic profile of anti-establishment voters, singling out older, white, less educated and economically disadvantaged individuals as the “left behind” of globalization. These disenfranchised voters, in their struggle to cope with rapid social, economic and cultural changes, turned their back on mainstream political parties (Goodwin and Heath, 2016; Hobolt, 2016; Clarke et al., 2017).

Next to these largely utilitarian perspectives, a second strand of literature focused on identity-driven motivations and the role of populist nationalism in the successful campaign for Brexit (Crewe and Sanders, 2020). The strong link between feelings of English national identity and Euroscepticism (Goodwin and Milazzo, 2017; Carl et al., 2019), combined with the fact that English voters see national identity and EU membership as conflicting (Kuhn, 2019), explain why “Brexit was made in England” (Henderson et al., 2017: 631; see also; Sobolewska and Ford, 2020). Compared to the predominantly economic focus of the previous two perspectives, these studies show that voters, especially males, the elderly and the low educated, begrudged the openness to immigration and progressive views of the cosmopolitan elites, and perceived EU membership as a cultural threat (Richards et al., 2018).

Unsurprisingly, given its issue salience in the referendum campaign, research has also focused on the role of immigration as a key driver of the Leave vote, one that is inextricably linked with the previous explanations (immigration posing both economic threats as well as cultural threats to “left behind” voters). Britons with highly negative attitudes about immigration were more likely to extol the benefits of Brexit in terms of immigration control, countering terrorism and British influence in world affairs and were more likely to have voted for Leave (Clarke et al., 2017; Henderson et al., 2017). Support for Leave was particularly strong in regions that experienced a faster rise in immigration from Eastern Europe between 2001 and 2011 (Becker et al., 2017; Colantone and Stanig, 2018). Goodwin and Milazzo (2017) found that people who perceived a rise in immigration were more likely to switch their vote intention from Remain to Leave in the weeks before the referendum; importantly, their analysis drew attention to voters’ desire to regain control over immigration as one of the strongest predictors of the Leave vote (see also Lord Ashcroft Polls, 2016).

Immigration control was a dominant theme in the campaign leading to the referendum and one that resonated well with the British electorate. Starting from the late 1990s, immigration was perceived as the most important issue facing the country by a rapidly increasing share of the British public. Concerns about immigration, as well as public demands for more restrictive policies in this domain, grew in parallel with a sharp rise in migration levels (Evans and Mellon, 2019; Ford et al., 2015). In particular, the EU-born population increased steadily following the decision by the British government to allow free labor market access to citizens of the eight Central and East European countries that joined the EU in 2004 (the Czech Republic, Estonia, Latvia, Lithuania, Hungary, Poland, Slovakia, and Slovenia), commonly referred to as A8 countries. EU nationals mostly migrated to the United Kingdom for work-related reasons and net inflows from A8 countries vastly exceeded predictions—some of these statistics also reflecting registrations from people already living as irregular migrants in the United Kingdom who legalized their status (Pollard et al., 2008). Although unintended, “from 2004 onwards, immigration to the United Kingdom became increasingly Europeanized” (Dennison and Geddes, 2018: 1139). Immigration from the accession countries was so substantial that it displaced commonwealth immigration as the largest source of migratory flows to the United Kingdom, with Poland becoming the most common country of birth of foreign-born residents (Evans and Mellon, 2019). The EU-born population reached 3.7 million in 2017 (Vargas-Silva and Fernandez-Reino, 2018); EU inflows peaked right before the referendum and, partly as a result of Brexit, decreased in the following years. A second generation of people with European backgrounds is also slowly emerging in Britain: in 2016, 12% of all newborns in England and Wales had at least one non-British European parent (Lessard-Phillips and Sigona, 2019).

The intensification of migration flows from the EU was not accompanied, however, by a trend towards a more inclusive, European identity in the British public. British Euroscepticism has old roots. Public opinion data show that, over the last 40 years, Britons’ sense of European identity has been consistently low compared to other EU member states (Heath and Spreckelsen, 2015; Carl et al., 2019). The clash between, on the one hand, the Europeanization of immigration and the nationally-oriented identity concerns of sections of the British public on the other, fostered increasingly Eurosceptic views. The relationship between concerns over immigration and disapproval of the EU substantially strengthened after the 2004 enlargement to the East (Evans and Mellon, 2019), paving the way to the electoral success of the anti-Europe, anti-immigration United Kingdom Independence Party (UKIP). At the same time, the British government proved ineffective at meeting public demands for a more restrictive immigration policy. Because free movement between EU member states is a fundamental right guaranteed by EU treaties, EU nationals living in a different member state than the one where they were born can be considered regional free-movers^1^ (Dennison and Geddes, 2018). Crucially, this distinction severely limited the ability of the British government to respond to public concerns in a thermostatic manner with stricter border controls or other restrictions to the immigration of EU nationals. This lack of policy responsiveness fueled discontent among British voters whose concerns about immigration inevitably remained unaddressed, and became the catalyst for UKIP’s rise in popularity (Ford et al., 2015; Evans and Mellon, 2019).

### From the Ballot Box to the Workplace: Brexit Support and Discrimination Against Applicants With EU Backgrounds in the British Labor Market

Why might support for Brexit translate into discrimination against Europeans in the labor market? Most of the research on discrimination using field experiments (the “gold standard” approach to identifying labor market discrimination) has focused on “visible” minorities largely from non-European former colonies of Britain such as India, Pakistan, Nigeria, and Jamaica (Heath and Di Stasio, 2019). However, the main theories used to explain discrimination against visible minorities can in principle apply equally to discrimination against non-British European job applicants too. Moreover, these theories of discrimination also parallel the utilitarian and cultural explanations that have been developed to account for Brexit and were summarized above, suggesting plausible links between the two phenomena.

The classic theories of the sources of discrimination have distinguished what are termed “statistical explanations” (which can be equated with the utilitarian explanations of support for Brexit) and “taste-based explanations” (which can be equated with the cultural and identity explanations of support for Brexit) of discriminatory behavior. Broadly speaking, the statistical theory of discrimination postulates that it will be rational for employers to discriminate if they have limited information about individual candidates’ likely productivity. In these circumstances, they may use statistical information about the likely productivity of the group from which the individual applicant comes. Thus if European applicants are seen as less productive on average because of lower levels of fluency in English, for example, then it is rational to prefer a native English-speaker to a European non-native speaker with a similar observed record and set of skills. The known average group characteristic is thus used as a proxy for the unobserved characteristics of the individual applicant in order to estimate the applicant’s productivity (Arrow, 1972; Phelps, 1972). In contrast, the taste-based account of discrimination postulates that an employer is prejudiced against certain classes of applicant and is therefore willing to hire a less productive worker from a non-stigmatized class of applicant in preference to a more productive one from a stigmatized class, even if that is irrational from a purely instrumental profit-maximizing perspective (Becker, 1957; Pager, 2016).

Both kinds of argument could apply in the case of discrimination against European applicants in general, and against East Europeans (post 2004 enlargement) applicants in particular. Thus, European applicants who were educated abroad will have foreign qualifications and may also have foreign work experience, which will mean that employers might expect them to take longer to adjust to a British work environment than will British applicants. Moreover, Eastern European migrants to Britain tend to be somewhat less qualified on average than the West European migrants, and to have lower-level work skills (Demireva, 2011). These considerations suggest that there could be some statistical discrimination against migrants from both groups, with higher levels of discrimination against the East Europeans than the West Europeans. To be sure, these considerations apply more to migrants than to the British-born second generation, but there is accumulating evidence that employers may not fully appreciate the difference between foreign-born and native-born migrants and that “poor language skills implicitly are assumed to be a problem when hiring ethnic minorities, regardless of generation” (Midtbøen, 2014: 1669). Consistent with this argument, several field experiments found that first and second-generation minority applicants experience similar levels of discrimination in hiring (Carlsson and Eriksson, 2017; Veit and Thijssen, 2021). In addition, Brexit means that there will be greater uncertainty about the future residence status of European job applicants in the United Kingdom, given the ending of Britain’s membership of the EU and the right of European citizens to work in Britain, thus meaning that their future expected productivity will be discounted to some extent. Indeed, a report commissioned by Deloitte and based on a crowdsourced sample estimated that nearly half of the surveyed highly skilled EU workers could leave Britain before 2022 (Deloitte, 2017). Recent estimates from the Office for National Statistics suggests this ‘Brexodus’ has already begun (ONS, 2021).

Turning to the taste-based theory of discrimination, theories of outgroup prejudice suggest that prejudice will increase the greater the cultural distance between the ingroup and outgroup. The history of Eastern Europe and its communist past (as well as the Orthodox Christian traditions in many European countries) suggests that there will be greater cultural distance and stronger symbolic boundaries in the case of the East Europeans. These expectations are in line with the evidence on attitudes towards different kinds of European migrants. This research has shown a clear hierarchy of positive and negative attitudes towards migrants from different origins, with the strongest positive attitudes for people of the same ethnic or racial group as the majority, followed by slightly (but significantly) less positive attitudes towards migrants from richer European countries (which we can broadly equate with EU12 countries), with more negative attitudes towards migrants from poorer countries in Europe (such as the 2004 accession countries), and more negative still against migrants from poorer countries outside Europe (broadly speaking the sources of visible minorities) (Heath and Richards, 2020).

On both utilitarian and cultural grounds, then, we would expect employers to discriminate against European migrants, with a higher level of discrimination against migrants from Eastern Europe. This expectation is also consistent with the limited evidence from field experimental studies that migrants of European background tend to face a fairly modest risk of discrimination in the British labor market (Heath and Di Stasio, 2019; Zwysen, Di Stasio and Heath, 2020) and in other Western societies more generally (Baert et al., 2017; Quillian et al., 2019; Thijssen et al., 2019).

Both utilitarian and cultural theories also imply that discrimination will tend to be greater in those areas of Britain where support for Brexit was stronger. The theory of statistical discrimination implies that employers with less experience of non-British workers will have greater uncertainty about their likely productivity and will therefore tend to discount their potential productivity (and will perhaps also in consequence employ incorrect stereotypes when making judgements about employability). The geographical distribution of ethnic minorities in Britain means that employers in London, where minorities constitute around half of the population, will have more experience of minorities whereas those in more strongly Brexit-supporting areas such as the North-East (with less than 10% minorities) will have least experience.

There may also be more direct links between the cultural and identity sources of support for Brexit and the taste-based sources of discrimination against foreign workers. While we should not exaggerate the importance of immigration as a driver of Brexit, it was certainly a major theme. Concerns about immigration also rose following the 2004 enlargement and the rapid increase of less-skilled migrants from Eastern Europe (Evans and Mellon, 2019). Finally, recent surveys of ethnic minorities found a statistically significant increase in fear of ethnic and racial harassment in the aftermath of the referendum (Nandi and Luthra, 2021) and significantly more episodes of self-reported discrimination among residents of areas with higher percentages of Leave voters than among residents of areas with fewer Leave voters (Frost, 2020).

## Data and Method

### Research Design

We rely on a field experiment on discrimination in hiring conducted in the British labor market as part of a larger cross-national project on ethnic discrimination (the GEMM project: Di Stasio and Lancee, 2020). Field experiments are a powerful method to detect discrimination in the hiring process as they rely on a comparison of employers’ responses to carefully matched bogus applications only differing in the ethnic or racial background of the candidate. The fieldwork began shortly after the Brexit referendum and continued until December 2017. We prepared fictitious CVs and cover letters and applied to 3195 jobs advertised through a very popular online portal managing over 160,000 job applications a day. To minimize the risk of detection and reduce burden for employers—who in field experiments are assessing fictitious applicants that they believe to be genuinely interested in the job—we opted for an unpaired (also known as unmatched) design and sent only one application per employer. Compared to paired designs, unpaired designs allow for easier implementation of multiple orthogonal treatments simultaneously and yield discrimination estimates that are less sensitive to the size of the applicant pools (Larsen, 2020).

Applicants were identical in terms of qualifications and work experience but differed in a number of characteristics. Innovatively, in the GEMM project over 30 different origin countries were randomly assigned to the applications, including European ones. This design allows us to test whether applicants originating from EU countries faced discrimination when applying for jobs, compared to white British applicants. In addition, we also randomly varied other characteristics across applications, namely: gender, religion, grades, and additional information on applicants’ past performance and social skills. As these characteristics are not the focus of this study, we do not discuss them further. We included them as controls in the analyses where appropriate and refer the reader to the codebook for more detailed information on the research design (Lancee et al., 2019a; Lancee et al., 2019b).

We responded to job openings advertised for any of the following six occupations: cook, store assistant, admin/payroll officer, receptionist, software developer, marketing/sales representative^2^. We tracked the responses received from employers and, in line with the standard protocol for field experiments, politely and immediately declined any invitation to job interviews or request to provide additional information.

### Variables

#### Dependent Variables

We regard average differences in callbacks between white British and EU applicants^3^ with otherwise identical characteristics as evidence of discrimination. We ran the same sets of analysis using two different operationalizations of callbacks. The first binary dependent variable, “any interest”, distinguishes between requests for additional information, communications of shortlisting decisions and invitations to an interview or a trial day (all coded as positive callbacks) and rejections or no responses (both coded as negative callbacks). The second binary dependent variable, ‘*interview*’, only includes direct invitations to interviews/trial days in the count of positive callbacks.

#### Independent Variables

The key variable of interest for our analysis is the country of origin of applicants. To signal applicants’ origin, we used foreign-sounding names (reported in the Supplementary Appendix Table A1). It is worth stressing that all applicants had received their education and training in Britain, had 4 years of domestic work experience in well-known British organizations and were fully qualified for the job they applied to^4^. This information was clearly signaled in the resume and in the cover letter and both documents were written without any spelling mistakes. In keeping with job application standards in the British context, country of birth was not explicitly mentioned in the resume. To reinforce the foreign-sounding name treatment, we explicitly referred to applicants’ origin country in the cover letter with the sentence: “note that although I have a (e.g. Italian) background all my education and training has been in Britain”. In half of the cases, we added that the applicant had moved to Britain at the age of six (i.e., first generation migrant). Furthermore, in the “skills section” of the resume, applicants of European origin always described themselves as bilingual and, next to English, listed their home-country language (e.g. “Bilingual English and Italian”). An example of the CV and of the cover letter used in the field experiment are included in the Supplementary Appendix (Figure A1).

In our analyses, we compared the callbacks received by the white British group (N = 725) with the callbacks received by applicants of European descent. We also split the group of European applicants into two sub-groups: applicants originating from EU12 countries (France, Germany, Greece, Ireland, Italy, Netherlands and Spain: N = 286) and applicants originating from Eastern Europe (Bulgaria, Poland and Romania: N = 100).^5^ Descriptives are presented in Table 1.

**TABLE 1:** Descriptive statistics.

	N applications	% Applications
**Background**		
White British	725	65.3
EU country	386	34.7
**EU background**		
EU12 country, *of which:*	286	10.6
France	41	
Germany	41	10.6
Greece	43	11.1
Ireland	34	8.8
Italy	38	9.8
Netherlands	45	11.7
Spain	44	11.4
Eastern European country, *of which:*	100	
Bulgaria	22	5.7
Poland	34	8.8
Romania	44	11.4
**Occupation**		
Cook	151	13.6
Payroll clerk	298	26.8
Receptionist	153	13.8
Sales representative and marketing analyst	182	16.4
Software developer	167	15.0
Store assistant	160	14.4
**Nuts1 regions**		
North East England	27	2.1
North West England	114	9.3
Yorkshire Humber	66	6.0
East Midlands	65	4.9
West Midlands	64	6.2
East of England	141	13.4
London	275	25.2
South East England	251	22.6
South West England	95	8.8
Wales	9	1.0
Scotland	4	0.4
**Callbacks**		
Any positive interest, *of which:*	258	23.2
White British	178	24.6
EU country	80	20.7
Invitation to interview, *of which:*	143	12.9
White British	97	13.4
EU country	46	11.9

Source: GEMM data, own calculations.

#### Controls

We included a series of controls in our models, in a step-wise fashion. First, we introduced a set of occupations dummies as employers might be more reluctant to hire minority applicants in customer-oriented jobs or in less tight labor markets, where supply of domestic labor is abundant. We also included dummies for contract type. Second, we controlled for all other characteristics, next to applicants’ origin, that were randomly varied in the design of the field experiment. Third, we controlled for the region where the job was located, using a set of dummies that correspond to the first-level NUTS regions of the United Kingdom (from the French Nomenclature d'Unités Territoriales Statistiques). This information was automatically recorded by the crawler when sampling jobs from the online portal and was only missing for one observation, which was excluded from the analysis. Fourth, we controlled for th*e time* when the application was sent, whether in the first semester after the Brexit referendum, the second or the third (which was also the last semester of our fieldwork). Finally, the crawler kept track of both the number of days that had passed since a job opening had been advertised and the number of applicants that had already applied at the time we sent an application. Based on this information, we computed the average number of people who applied, daily, to any given job. While it is of course possible that interested job seekers applied to the same jobs through other channels, we consider this variable a reasonable proxy of competitiveness.

### Estimation Strategy

As our dependent variables are binary, and in keeping with common practices in the field experimental literature, we ran linear probability models (LPMs) with robust standard errors. We prefer linear probability models as they are more intuitive to interpret than logit or probit models, particularly in relation to interaction effects. LPM coefficients are closely related to average marginal effects derived from logit or probit models and can be easily compared across models, contrary to odds ratios and coefficients derived from nonlinear probability models (Breen et al., 2018; Gomila, 2020). To test for differences in levels of discrimination across regions, we also ran two-step multilevel models, a technique to deal with nested data that is especially recommended when the number of clusters at the macro level is low and the focus is on cross-level interactions (Heisig, Schaeffer and Giesecke, 2017). This is exactly the case in our study, as we have a low number of clusters (i.e., NUTS1 regions) and we are interested in whether the level of discrimination faced by European applicants was more severe in regions where support for Leave was stronger.

In a first step, we estimated region-specific regressions, limiting our focus to NUTS1 regions in which we had sent a minimum of 50 applications (thus excluding North East England, Scotland and Wales from this analysis)^6^. Given the relatively smaller number of observations within each region, we opted for parsimonious models that, next to including the origin-country dummies, only controlled for occupations and competitiveness. These controls are important because specific jobs might be concentrated geographically and some regions might be more dynamic than others and have a tighter regional labor market. We saved the estimates of interest—i.e., the beta coefficients for the origin-country dummies and their standard errors—for further analysis. These betas reflect the size of the callback gap between white British applicants and applicants of European origin: the more negative the gap, the stronger the level of discrimination faced by European applicants. In a second step, coefficient estimates from the first step became outcome variables in a cluster-level (in this case, a region-level) regression, also known as “slopes-as-outcomes regression”, where the key parameter of interest was the association between the region-specific callback gaps and the share of Leave support within each NUTS1 region. Because the dependent variable in this second step was itself a coefficient that had been estimated with a degree of imprecision that varies across NUTS1 regions, we applied feasible generalized least squares (FGLS) using the edvreg command in Stata, which weights down unreliable estimates in the cluster-level regression (Lewis and Linzer, 2005).

We checked the robustness of our findings with a different model specification. We ran a multilevel random-slope model using restricted maximum likelihood (REML) estimation and the Kenward and Roger approximation (Kenward and Roger, 1997). With this modelling strategy, the test statistic of the cross-level interaction term was computed based on the t-distribution, which is recommended in order to avoid anti-conservative p-values and confidence intervals for hypothesis testing (Elff et al., 2021). Results were in line with those obtained with the two-step estimation, even though the cross-level interaction was only significant at *p* < 0.1 for the interview variable (as our hypothesis is directional, the one-sided test of the hypothesis would still be statistically significant at conventional levels). Finally, we also relied on visualization to inspect the contextual variation in discrimination, as shown below. We agree with Bowers and Drake’s (2005: 323) observation that “when a result does emerge from visualization, it does hit the audience between the eyes, and thus may be as compelling as many asterisks beside a coefficient in a table”, particularly when dealing with a limited number of clusters. This visualization also reveals that the association between the Brexit vote and the discrimination coefficient is driven by the London region^7^.

## Results

### Are Job Applicants of European Origin Discriminated Against in the British Labor Market?

We start the presentation of results by comparing the callbacks received by the white British group with those received by European applicants as a whole (including both EU12 and Eastern European applicants). With regard to our less strict callback indicator (any interest from employers), about one in four applicants from the white British group (24.55%) was called back. This was the case for about one in five European applicants (20.73%). The callback ratio (1.18) indicates that European applicants had to send about 20 percent more applications than the majority group to receive a comparable number of callbacks. This callback ratio is close to the upper bound of the interval found for White minorities in a meta-analysis of British field experiments conducted since the end of the 1960s (Heath and Di Stasio, 2019). When differentiating between EU12 and Eastern European applicants, the callback ratios are 1.17 and 1.23, respectively. Overall, differences between the two groups are negligible. For the stricter callback indicator (invitations to interview), the callback ratio is 1.12 for the group of European as a whole, 1.16 for EU12 applicants and 1.03 for Eastern Europeans. To put these findings in perspective, the callback ratio for non-white minorities (black Africans, Caribbeans, Chinese and South Asians), also included in the field experiment but not the focus of this study, was 1.91 (1.77 when using the stricter callback measure), meaning that they had to apply almost twice as often as the white British to receive a comparable number of positive responses from employers. A two-sided test of proportions indicates that differences in callback rates between European applicants and applicants from visible minorities are statistically significant (*p* < 0.01).

Table 2 shows the callback ratios for each occupation separately. The disadvantage faced by EU applicants is concentrated in non-graduate jobs, especially in hospitality and administration. In high-skilled jobs, EU applicants were even positively discriminated, even though this advantage is not statistically significant. Somewhat unexpectedly, we found evidence of discrimination in jobs that required little customer contact but not in customer-facing jobs.

**TABLE 2:** Callbacks, by occupation.

Occupation	EU backgrounds	EU12	Eastern EU
Cook	**1.56**	1.42	2.01
Payroll clerk	**2.14**	1.86	4.12
Receptionist	1.14	1.29	0.78
Sales representative and marketing analyst	0.87	0.79	1.35
Software developer	0.92	1.01	0.76
Store assistant	1.04	0.96	1.28
Graduate level	0.89	0.91	0.83
Below graduate level	**1.43**	1.37	1.62
Client-facing	0.99	0.96	1.13
Not client-facing	**1.34**	1.34	1.32

Source: GEMM data, own calculations.

The breakdown by single occupation for Eastern European applicants is only indicative, as the N per occupation is very low (<25). In the second column, bold numbers refer to occupations where EU nationals are significantly discriminated (*p* < 0.05), according to a two-sample test of proportions. We did not run these tests for the two European groups separately, given the lower N.

The linear probability models presented in Table 3 test whether the callback gaps between groups remain statistically significant after controlling for occupations, job and applicant characteristics (contract type, and the other treatments that randomly varied in the field experiments), NUTS1 regions, time of the application and labor competition. As reported in model 1, the gap between white British applicants (the reference category) and European applicants is about eight percentage points to the disadvantage of the latter, and statistically significant, even after adding all controls. Model two shows a slightly more negative gap in callbacks for Eastern European than for EU12 applicants, even though the difference between these two groups is not statistically significant. In other words, we cannot reject the hypothesis that both groups are discriminated against in the British labor market to a similar degree. Model three confirms that the gap is still present and statistically significant when considering the stricter callback measure: the probability to be invited for a job interview is six percentage point lower for EU applicants; however, this disadvantage is statistically significant only in the case of EU12 applicants, as can be seen in model 4 (here too, we cannot reject the hypothesis that both groups are discriminated against in the British labor market to a similar degree; indeed, the point estimates for the two groups are very similar). When running logistic regressions instead of LPMs, we obtained comparable results.

**TABLE 3:** Callback gaps between white British applicants and those with EU backgrounds (linear probability models).

	Any interest		Invitation to interview	
	**M1**	**M2**	**M3**	**M4**
EU-country origin	−0.079**		−0.060**	
	(0.031)		(0.024)	
Ref. White British				
EU12 background		−0.074**		−0.064***
		(0.033)		(0.024)
Eastern EU background		−0.097**		−0.049
		(0.049)		(0.039)
Constant	0.500***	0.500***	0.428***	0.428***
	(0.063)	(0.063)	(0.054)	(0.054)
N applicants	1096	1096	1096	1096
R-squared	0.086	0.086	0.085	0.085

Robust standard errors are in parentheses.

*** p <.01, ** p <.05, * p <.1.

Source: GEMM data, own calculations.

EU12 countries: France, Germany, Greece, Ireland, Italy, Netherlands, Spain. Eastern EU countries: Bulgaria, Poland, Romania.

Models include controls for: occupations, type of contract, applicants’ characteristics, nuts1 regions, competitiveness (daily n. applicants/job), time dummies.

The full models, with step-wise inclusion of the control variables, can be found in the Supplementary Appendix (Tables A2–A5). With regard to the control variables, the probability to receive a callback was lower for jobs as payroll clerk, store assistant, receptionist and sales representative than for cooks, and this is partly due to differences across occupations in labor supply: the gaps in callbacks decrease, but do not disappear, after controlling for our competitiveness proxy. In additional models, we also included a control for the degree of urbanization (measured at the NUTS2 level, and distinguishing between predominantly urban, mixed and predominantly rural areas) and results were stable. We also tested formally whether discrimination was more severe in non-graduate occupations, but the interaction was not statistically significant. The models also show that applicants were less likely to receive a positive response if they applied for jobs that were in high demand (more than ten applicants, on average, per day) or in less dynamic regions than the Greater London area, particularly the North and South West, East of England and Scotland.

Finally, across models, applicants who stated that they had moved to Britain at the age of six (by implication foreign-born migrants) were more likely to receive a callback than were applicants whose letters did not specify whether they were migrants or second-generation. When directly comparing these groups with the white British group, it appears that only the latter were discriminated against. While this result might seem surprising, it should be interpreted with caution, as country of birth was not explicitly mentioned in the job application. It is possible that employers considered applicants who wrote in the cover letter that they had been in Britain since the age of six as long-term residents while perceiving applicants who only wrote that they had obtained all relevant education and training in Britain as migrants who moved to the country at a later stage (e.g. late childhood). We recognize that the signal of migration status was not ideal, but we preferred to avoid mentioning country of birth in the application for reasons of ecological realism. (When preparing the study we found that it was very rare for genuine applicants with foreign-sounding names to specify their country of birth in the curriculum.)

### Is Discrimination Stronger in Regions With a Larger Support for Leave Among British Voters?

We now move to the second part of our analysis, and examine whether the level of discrimination faced by job applicants of European origin is stronger in NUTS1 regions where a larger share of the electorate voted for Leave. First, in Table 4, the sub-group analysis indicates that employers did not discriminate against European migrants in the immediate aftermath of the referendum. We split the sample into three groups, according to the date when the job application was sent (during the first semester after the referendum, the second or the third). While in the first semester European applicants are treated on a par with the white British majority, in the second semester the gap in callbacks between the two groups widens to a substantial 11 percentage point difference, which remains rather stable in the last semester of fieldwork.

**TABLE 4:** Discrimination against applicants with EU backgrounds, by post-Brexit semester.

	M1: 1^st^ semester		M2: 2^nd^ semester		M3: 3^rd^ semester	
	Any	Interview	Any	Interview	Any	Interview
EU-country origin	0.010	0.012	−0.108**	−0.105***	−0.109**	−0.07**
	(0.08)	(0.074)	(0.055)	(0.029)	(0.047)	(0.035)
_cons	0.478***	0.463***	0.522***	0.337***	0.523***	0.380***
	(0.137)	(0.13)	(0.103)	(0.077)	(0.088)	(0.075)
Observations	214	214	391	391	491	491
R-squared	0.121	0.141	0.113	0.110	0.126	0.100

Robust standard errors are in parentheses.

*** p <.01, ** p <.05, * p <.1; two-sided.

Source: GEMM data, own calculations.

Models include controls for: occupations, type of contract, applicants’ characteristics, nuts1 regions, competitiveness (daily n. applicants/job).

While we can only speculate about these differences across semesters, it is interesting to note that it is during this second semester (namely on March 29, 2017) that Article 50 was invoked, i.e., the formal procedure through which the United Kingdom notified the European Council of its intention to withdraw from the EU and that led to the start of the withdrawal negotiations. Another possible explanation for this pattern of findings is that employers gradually found themselves amidst growing uncertainty and refrained from hiring EU applicants while waiting for clearer indications on how to plan their post-Brexit recruitment strategies. Consistent with this view, according to a survey conducted by the Chartered Institute of Personnel and Development (CIPD), a professional association for human resource management professionals, more than half of employers felt that they were left completely in the dark about the Government’s immigration proposals and did not have sufficient information about the Government’s white paper on immigration (CIPD, 2019).

Moving to our second hypothesis of stronger discrimination in more pro-Brexit areas, Table 5 reports a breakdown of the callback ratios by NUTS1 regions, with the regions being ranked according to the share of Leave support. It is interesting to note that while the ratios are even slightly in favor of European applicants in the Greater London area, where most people voted Remain, applicants from the white British group were strongly preferred in the North and South West and in Yorkshire and Humber. More surprising are the callback ratios recorded in the Midlands, given the relatively larger share of Leave voters in those areas.

**TABLE 5:** Callbacks, by nuts1 regions.

Nuts1 regions	% Leave vote	Any interest			Invitation to interview			N of sent applications
		White british	EU back-grounds	Callback ratio	White british	EU back-grounds	Callback ratio	
West Midlands	59.3	21.9	21.7	1.0	9.8	13.0	0.7	64
East Midlands	58.8	19.1	30.4	0.6	14.3	13.0	1.1	65
Yorkshire Humber	57.7	28.2	7.4	3.8	7.7	0.0	_	66
East of England	56.5	24.1	16.7	1.4	14.9	9.3	1.6	141
North West England	53.7	20.3	12.5	1.6	8.1	7.5	1.1	114
South West England	52.6	21.9	9.7	2.3	7.8	3.2	2.4	95
South East England	51.8	26.9	22.4	1.2	11.4	10.5	1.1	251
London	40.1	25.8	28.9	0.9	19.1	21.6	0.9	275
**Total sample**	**51.9**	**24.5**	**20.7**	**1.2**	**13.4**	**11.9**	**1.1**	**1111**

Source: GEMM data, own calculations. We only retained nuts1 regions in which more than 50 applications were sent (as a result, applications sent in North East England, Scotland and Wales were excluded from these calculations). The callback ratio reflects the relative advantage of white British applicants over EU nationals; EU countries of origin: Bulgaria, France, Germany, Greece, Ireland, Italy, Netherlands, Poland, Romania, Spain.

To formally test our hypothesis, we first ran linear probability models for each one of the NUTS1 regions where we sent at least 50 applications and stored the beta coefficients associated with the European group’s dummies, and their standard errors. In a second step, we regressed these estimated coefficients on the share of the Leave vote in the region following the procedure described above (see section 3.3). While our hypothesis was not supported for the group of Europeans as a whole, we found that, after controlling for occupations and labor competitiveness, employers discriminated more strongly against EU12 applicants in NUTS1 regions characterized by a higher share of Leave voters. Results of the two-step estimation are reported in Table 6. The constant refers to the probability to be called back for European applicants, relative to the white British group, when the Brexit vote is held at its mean level, and is negative in all models. Based on these results, we calculated that the predicted callback gaps between the two groups across British regions range from nine percentage points to the disadvantage of EU12 applicants to five percentage points in favor of EU12 applicants, depending on the support for Brexit recorded in the region. Results are similar for the two callback indicators, although this difference is statistically significant at *p* < 0.05 only in the model using invitations to interviews as the dependent variable, while it is marginally significant for the less strict callback indicator. Interestingly, no association between the referendum results and the size of callback gaps was found for Eastern European applicants, an issue we come back to in the discussion.

**TABLE 6:** Cross-regional variation in the gap in callbacks between white British applicants and those with EU backgrounds: two-step FGLS estimation.

	Any interest						Invitations to interview					
	EU		EU12		Eastern EU		EU		EU12		Eastern EU	
	Incl. London	No London	Incl. London	No London	Incl. London	No London	Incl. London	No London	Incl. London	No London	Incl. London	No London
% Leave (centered)	−0.004	0.005	−0.007*	−0.001	0.005	0.022	−0.005	−0.005	−0.008**	−0.005	0.002	0.007
	(0.005)	(0.013)	(0.003)	(0.009)	(0.01)	(0.025)	(0.003)	(0.007)	(0.003)	(0.007)	(0.005)	(0.01)
Constant	−0.046	−0.072	−0.043*	−0.056*	−0.051	−0.102	−0.031	−0.049*	−0.042**	−0.049*	−0.042	−0.058
	(0.032)	(0.048)	(0.020)	(0.028)	(0.061)	(0.095)	(0.017)	(0.023)	(0.016)	(0.023)	(0.025)	(0.039)
N regions	8	7	8	7	8	7	8	7	8	7	8	7
R-squared	0.098	0.025	0.472	0.006	0.042	0.134	0.281	0.002	0.504	0.083	0.029	0.09

Standard errors are in parentheses.

*** *p* < 0.01, ** *p* < 0.05, * *p* < 0.1.Source: GEMM data, own calculations.Dependent variable estimated from separate region-specific linear probability models in the first step. Results are unchanged if North East England is retained in the analysis.

**FIGURE 1:**
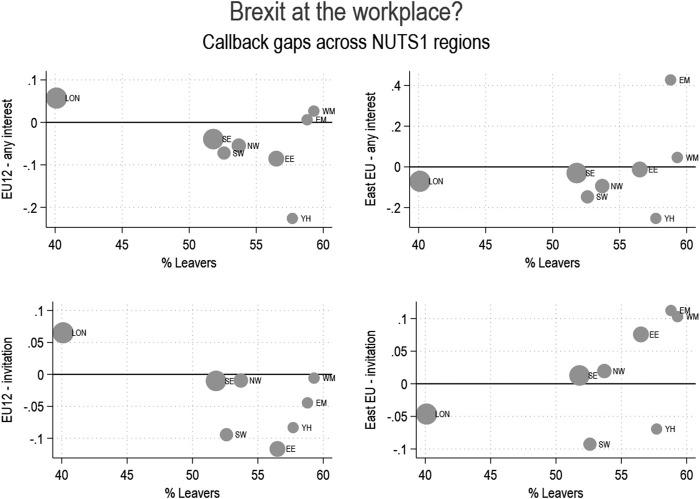
Discrimination and the Brexit vote.

To aid interpretation, we also include a visual presentation of these findings in Figure 1, which clearly shows that the association found in Table 6 is driven by the much more favorable treatment afforded to EU12 applicants by employers located in the Greater London area. Indeed, the association shown in Table 6 turns non-significant and negligible in size after removing from the analysis applications for jobs located in London.

**FIGURE 2:**
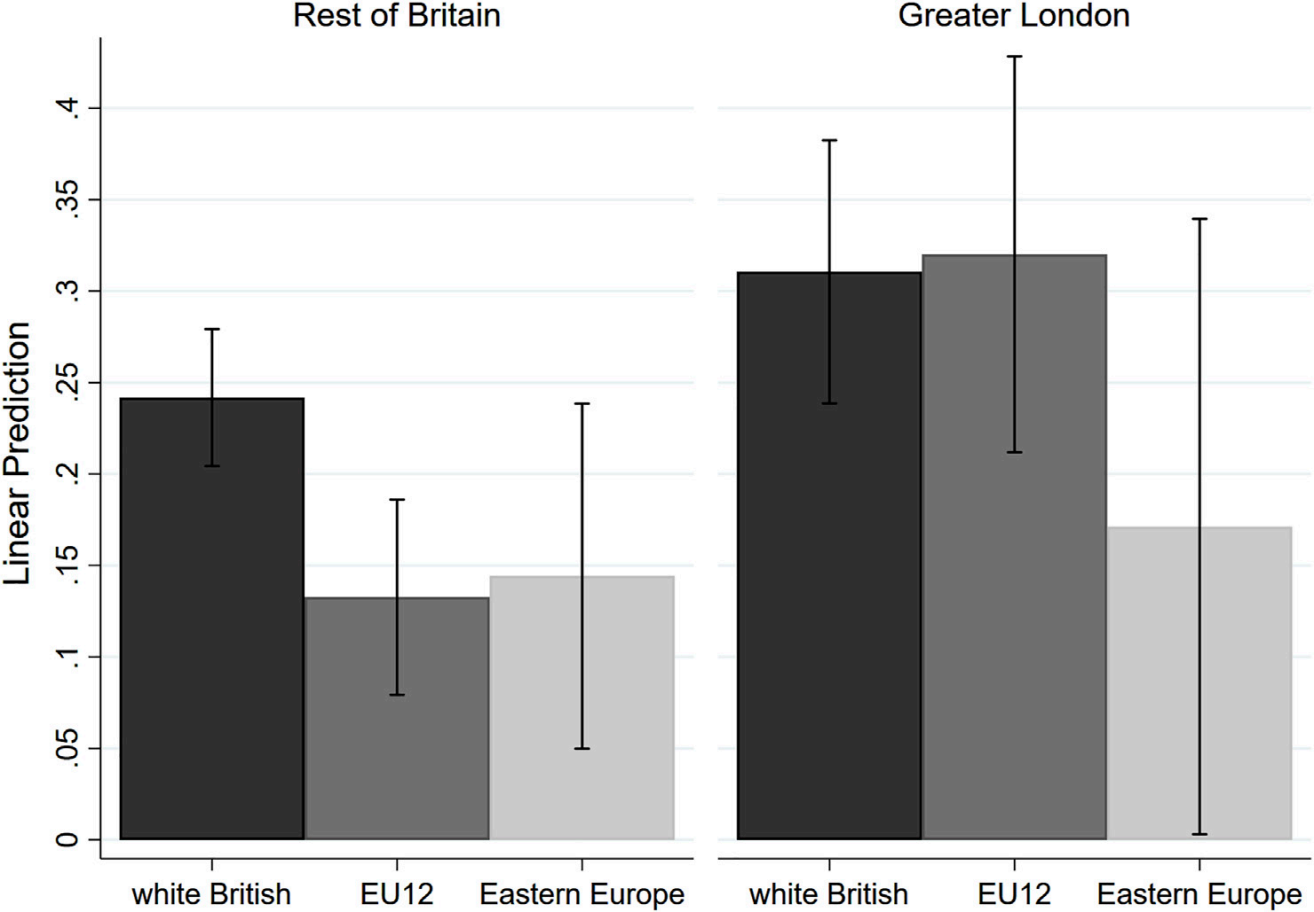
Predicted callbacks (any interest), by group: London vs. rest of Britain.

Finally, we zoom in on the Greater London area, a region with a much stronger support for Remain and known for its cosmopolitanism and international orientation. Figure 2 plots the results of a linear probability model identical to that reported in Table 3 (model 2) but this time including an interaction term between the EU group dummies and a dummy variable differentiating between Greater London and the rest of Britain. The interaction (marginally significant, *p* < 0.1) indicates that EU12 applicants were treated on par with the white British group in the London area; Eastern European applicants, on the other hand, do not seem to benefit from London’s cosmopolitanism and their callback gaps are rather similar across regions. Admittedly, contrasts between predicted probabilities reveal no statistically significant differences in the callbacks received by the Eastern European group and the white British group in the Greater London area, but this is very likely due to the small number of Eastern Europeans included in the study, which leads to very large confidence intervals, as can be seen from the figure. While the predicted callbacks are very similar for the two European groups in the regions outside of London, in the Greater London area the probability to be called back is nearly twice as large for EU12 applicants as it is for Eastern Europeans. To use Favell’s (2008) metaphor, London proves to be a truly *Eurocity*, but a selective one (see also King et al., 2016): the figure suggests that only EU12 applicants enjoyed a boost in their employment chances when applying to jobs in and around London.

## Discussion and Conclusion

In this study, we set out to test whether applicants with EU backgrounds, in the aftermath of the Brexit referendum and the wave of populist nationalism that accompanied it, faced a hostile environment when applying for jobs in Britain. We relied on a correspondence test conducted between August 2016 and December 2017 and randomly varying applicants’ background across employers to capture discrimination in hiring. An innovative feature of our research design was the inclusion of a large number of European origin countries, which allowed us to compare the callbacks received by EU12 applicants and Eastern Europeans with those received by the white British group. We further exploited regional variation in callback gaps to test whether employers discriminated more strongly against EU applicants in regions with a higher percentage of Leave voters and where nationalist and anti-European sentiments were likely to be stronger.

The findings indicate that, overall, employers discriminated against EU applicants from both groups, and to a similar degree, although the disadvantage faced by EU applicants was relatively modest if compared with that experienced by visible non-European minorities from South Asian, African and Caribbean descent. While our preliminary analysis suggested that EU12 applicants were more severely discriminated by employers in regions with a stronger support for Leave in the referendum, further analysis showed that this result was due to the pull of London, where EU12 applicants were treated on a par with the white British group. Eastern European applicants, on the other hand, did not appear to benefit from this more cosmopolitan environment. This “selective cosmopolitanism” cannot be due to employers’ reluctance to hire Europeans out of legal and administrative uncertainties, as both groups were facing the same legislative framework. Surprisingly, the percentage of Leave voters in the region was not associated with employers’ tendency to discriminate against Eastern Europeans, although we should remember that we had only 100 East European cases in the dataset compared with 286 EU12 cases.

At any rate, in the case of EU12 applicants, there is a striking contrast between London and the rest of the country, both with respect to Leave voting and to discrimination. Thus among Londoners only 40.1 percent voted Leave, contrasting with percentages ranging from 51.8 to 59.3 in the other English regions. Correspondingly, as Figure 1 shows, London stands out with positive discrimination in favor of EU12 applicants contrasting with the more or less negative rates of discrimination in most of the other regions, forming a cluster in Figure 1 on or below the x axis (representing equal treatment) and quite separate from the position of London.

As well as having a much lower percentage voting Leave, London also stands out as having more positive attitudes to immigration, much less English nationalism and a much larger immigrant population than do the other regions: in 2019, 35% of London residents were born abroad with the percentages ranging from 14 to 6 in the other English regions (House of Common Library, 2021). A similar pattern applies to Europeans: according to the 2011 census, 14.9% of London residents belonged to the “white other” ethnic group—predominantly Europeans—while in other regions the figures ranged from 1.7 percent to 5.5 percent (Race Disparity Unit, 2021). Contact theory in social psychology provides a plausible mechanism whereby the hyper-diversity of London generates more occasions to get in contact with Europeans, a more cosmopolitan worldview among Londoners and hence fewer people with tastes for discrimination (Allport, 1954; Pettigrew, 1998).

To be sure, London also stands out from the other regions in its economic performance (Blackaby et al., 2020), and indeed this economic dynamism is likely to be a major reason for its attractiveness to migrants. We cannot therefore discount the relevance of economic factors as the ultimate causes of London’s cosmopolitanism. At the same time, our controls for the tightness of the labor market strongly suggest that economic considerations are not the proximate causes of the contrasting levels of discrimination in London and in the regions outside London. Regional analyses by Blackaby and colleagues suggest that London may provide a different cultural context from the other regions of England. In every English region except London, concerns about immigration were strong and highly significant predictors of voting Leave, whereas in London these concerns were small and non-significant (Blackaby et al., 2020). In turn, this suggests that cultural and identity-based factors, rather than utilitarian ones, may be the main driver of London exceptionalism with regard to discrimination. A recent study of Norwegian employers concluded that “In societies where stereotypes are deeply embedded, we suggest employers are likely to resort to stereotype-based reasoning. In contexts where attitudes to specific groups are not as deeply embedded, employers are more likely to make hiring decisions based on experience-based reasoning. In lieu of strong stereotypes, direct experience becomes a more relevant source of information” (Birkelund et al., 2020, p.521). London might well provide precisely this kind of cultural milieu.

We must however acknowledge the limitations of this study. As with previous studies of the relationship between public opinion and rates of discrimination, we have been able to show only a correlation between the two variables. London was also the only region in our sample where voters were predominantly pro-Remain (we only sent a handful of applications to jobs in Scotland, which we dropped from the two-step analysis). A more powerful research design would entail interviews directly with the gatekeepers in firms which had participated in the field experiments in order to determine whether gatekeepers who were more prejudiced or who had more negative stereotypes of minorities were also more likely to make discriminatory decisions and to have voted for Leave.

We also acknowledge that the findings for East European job applicants do not fit with the EU12 results. This could be because East European migrants in practice have tended to enter agricultural, skilled manual and service work positions rather than the more professional occupations of EU12 migrants, especially in London. As a result, employers might not have as much familiarity and experience with this group of European migrants. But we must also note that our study is underpowered for a comparison of regional differences in the treatment of EU12 and Eastern European job applicants, with large confidence intervals and therefore an inability to rule between alternative hypotheses.

Finally, a more nuanced analysis of the relationship between discrimination and the Brexit vote would require a more detailed regional breakdown. Based on the information retrieved by the crawler, we could only distinguish between NUTS1 regions, which masks considerable within-region variation in voters’ support for Leave and, possibly, in levels of discrimination.

## Data Availability

The datasets presented in this study can be found in online repositories. The names of the repository/repositories and accession number(s) can be found below: The data used for this study have been deposited at DANS (Data Archiving and Network Services), the Dutch national repository for research data, and can be cited as: Lancee, B; Birkelund, G.E.; Coenders, M; VD; Fernandez Reino, M; Heath, A; Koopmans, R; Larsen, E.N.; Polavieja, J; Ramos, M; Thijssen, L; Veit, S; Yemane, R (2021): The GEMM Study: A Cross-National Harmonized Field Experiment on Hiring Discrimination. DANS. https://doi.org/10.17026/dans-zrz-m9cm. The NUTS1 code of the job applications have been manually coded by the first author, who can provide this information upon request.

## Footnotes

1 The status of EU nationals is ambiguous from a legal perspective, too: the law distinguished between persons who are or are not subject to immigration control. As long as the United Kingdom was part of the EU, EU-born nationals were not subject to immigration control, even though they were commonly portrayed as migrants (Anderson and Blinder, 2019).

2 We also applied to job openings in blue-collar occupations (electricians and plumbers), but the number of advertised jobs in these occupations was quite low during our fieldwork (at least on the online portal from which we sampled jobs). As we were only able to send 48 applications in total for jobs as electricians and plumbers (while we applied to more than 500 jobs, on average, in each of the other six occupations), we have dropped them from this analysis. Note that the main results do not change; if anything, when retaining these observations, the two-step analysis shows, for both types of callbacks (any sign of interest from employers and invitations to job interviews), a statistically significant association (*p* < 0.05) between the white British/EU12 applicants callback gap, on the one hand, and the Leave support in the Brexit referendum, on the other hand. In other words, the results we present in the following are more conservative.

3 We use the term EU applicants or EU backgrounds as a shorthand to refer to our fictitious respondents with European-sounding names. The application materials did not actually specify whether the applicants were nationals in the sense of formal citizenship but only that they had, for example, an Italian background.

4 We kept track of whether specific certificates or work experience were required and dropped those cases in which our applicants were under- or overqualified.

5 EU12 countries refer to the 12 EU countries before the 1995 enlargement while the three Eastern European countries joined the EU after the 2004 enlargement. To improve comparability, we excluded from the analysis eight applicants of Bulgarian origins and Muslim faith. All applicants included in the analyses were either Christian or did not mention their religious affiliation (which was proxied by volunteer work in a religious association) in the application. Our sample also excludes applicants originating from non-EU countries, which were also included in the original field experiment design. After dropping those who applied for jobs as plumbers or electricians, those who were not fully qualified, those who originated from outside the EU, one observation with no information about the location of the job, our sample consists of 1111 applicants.

6 Results are unchanged when using a minimum of 25 applications, thus retaining North East England for the second stage regression.

7 Ideally, we would consider a more disaggregated level of analysis than the NUTS1. While we could retrieve information on the NUTS2 level for all jobs outside London, we could not differentiate between inner and outer London as this information is not available in the dataset. This is unfortunate, given that one fourth of our sample consists of applications to jobs in the Greater London area. As a robustness check, we also estimated a multilevel model with a random slope and a cross-level interaction between the Leave support in the NUTS2 region and the origin dummies, while merging the inner and outer London regions to the NUTS1 level. The results are comparable.
