# Supplementary material for: London Calls? Discrimination of European Job Seekers in the Aftermath of the Brexit Referendum
Source: Front Sociol. 2021 Dec 22;6:737857. doi: 10.3389/fsoc.2021.737857 (PMC8727760; doi:10.3389/fsoc.2021.737857)
Supplement: Supplementary file 1 [file Table1.pdf]

APPENDIX

Table A1. Names and surnames randomly assigned to the job applications.

	National origin signaled in job applications as:			
National origin	Male first names	Female first names	Surnames	Language skills
Bulgaria	Zlatan	Nevena	Dimitrov/a	Bilingual English and Bulgarian
France	Guillaume	Claire	Durand	Bilingual English and French
Germany	Paul	Lisa	Schneider	Bilingual English and German
Greece	Giorgos	Konstantina	Papadopoulos/u	Bilingual English and Greek
Ireland	Erin	Brendan	O’Brien	English
Italy	Francesco	Valentina	Marino	Bilingual English and Italian
Netherlands	Jeroen	Maaike	De Vries	Bilingual English and Dutch
Poland	Marek	Michalina	Kowalski	Bilingual English and Polish
Romania	Andrei	Dana	Popescu	Bilingual English and Romanian
Spain	Alvaro	Alba	Martínez García	Bilingual English and Spanish
United Kingdom	James	Emily	Robinson	English

Table A2. Discrimination of European applicants (any interest from employers). Full table.

	M1	M2	M3	M4	M5
EU-country origin	-.043*	-.085***	-.083***	-.079**	-.079**
	(.026)	(.031)	(.031)	(.031)	(.031)
Occupation (ref. cook)					
Payroll clerk	-.268***	-.26***	-.268***	-.23***	-.229***
	(.045)	(.045)	(.045)	(.051)	(.051)
Receptionist	-.256***	-.254***	-.266***	-.216***	-.216***
	(.050)	(.050)	(.049)	(.056)	(.056)
Sales representative	-.211***	-.212***	-.213***	-.187***	-.188***
	(.050)	(.050)	(.050)	(.052)	(.053)
Software developer	-.067	-.064	-.06	-.054	-.054
	(.055)	(.055)	(.055)	(.056)	(.056)
Store assistant	-.172***	-.173***	-.175***	-.144***	-.144***
	(.052)	(.053)	(.053)	(.055)	(.056)
Contract (ref. full time)					
Part time	-.065	-.061	-.058	-.066	-.066
	(.040)	(.041)	(.040)	(.040)	(.041)
Flexible	.001	-.001	.004	.017	.016
	(.056)	(.056)	(.055)	(.055)	(.055)
Not specified	-.036	-.034	-.037	-.031	-.029
	(.045)	(.044)	(.044)	(.045)	(.045)
Other treatments:					
First generation		.077*	.075*	.071*	.072*
		(.041)	(.041)	(.041)	(.041)
Female		.056**	.06**	.058**	.058**
		(.025)	(.025)	(.025)	(.025)
Higher grade		-.038	-.033	-.033	-.033
		(.025)	(.025)	(.025)	(.025)
Performance signal		.030	.029	.034	.034
		(.025)	(.025)	(.025)	(.025)
Warmth signal		-.015	-.013	-.012	-.013
		(.025)	(.025)	(.025)	(.025)
Christian religion		-.019	-.022	-.022	-.022
		(.025)	(.025)	(.025)	(.025)
Nuts1 (ref. London)					
North East England			.056	.004	.002
			(.094)	(.095)	(.095)
North West England			-.12***	-.142***	-.143***
			(.045)	(.047)	(.047)
Yorkshire Humber			-.080	-.101*	-.101*
			(.056)	(.058)	(.058)
East Midlands			-.062	-.084	-.084
			(.057)	(.06)	(.06)
West Midlands			-.067	-.109*	-.109*
			(.057)	(.06)	(.06)
East of England			-.076*	-.104**	-.104**
			(.043)	(.045)	(.045)
South East England			-.032	-.058	-.058
			(.038)	(.041)	(.041)
South West England			-.119**	-.154***	-.154***
			(.046)	(.05)	(.05)

Wales			-.043	-.045	-.043
			(.135)	(.147)	(.148)
Scotland			-.298***	-.308***	-.305***
			(.083)	(.084)	(.085)
Average daily competition (ref. <1)					
1 to 2				-.034	-.033
				(.031)	(.031)
2 to 5				-.040	-.040
				(.045)	(.045)
5 to 10				-.056	-.056
				(.049)	(.049)
More than 10				-.141**	-.14**
				(.056)	(.056)
Time dummies (ref. 1st semester)					
2nd semester					-.012
					(.036)
3rd semester					-.013
					(.036)
Constant	.432***	.425***	.476***	.490***	.500***
	(.042)	(.051)	(.055)	(.058)	(.063)
N applicants	1111	1111	1111	1096	1096
R-squared	.060	.071	.083	.086	.086

Robust standard errors are in parentheses.
*** p<.01, ** p<.05, * p<.1

Source: GEMM data, own calculations.
Dependent variable: any interest from employers.
EU countries include: Bulgaria, France, Germany, Greece, Ireland, Italy, Netherlands, Poland, Romania, Spain.

Table A3. Discrimination of European applicants (any interest from employers): a comparison of EU12 and Eastern European migrants. Full table.

	M1	M2	M3	M4	M5
Origin (Ref. White British)					
EU12 origin	-.037	-.076**	-.076**	-.074**	-.074**
	(.028)	(.032)	(.032)	(.033)	(.033)
Eastern EU origin	-.063	-.112**	-.105**	-.096**	-.097**
	(.042)	(.049)	(.049)	(.049)	(.049)
Occupation (ref. cook)					
Payroll clerk	-.269***	-.261***	-.268***	-.231***	-.23***
	(.045)	(.045)	(.045)	(.051)	(.051)
Receptionist	-.257***	-.256***	-.267***	-.217***	-.217***
	(.05)	(.05)	(.05)	(.056)	(.056)
Sales representative	-.212***	-.213***	-.214***	-.188***	-.189***
	(.05)	(.05)	(.05)	(.052)	(.053)
Software developer	-.067	-.064	-.06	-.054	-.054
	(.055)	(.055)	(.055)	(.056)	(.056)
Store assistant	-.172***	-.173***	-.175***	-.144***	-.144***
	(.052)	(.053)	(.053)	(.055)	(.056)
Contract (ref. full time)					
Part time	-.065	-.061	-.058	-.066	-.066
	(.04)	(.041)	(.04)	(.04)	(.041)
Flexible	.001	0	.005	.017	.017
	(.056)	(.056)	(.055)	(.056)	(.055)
Not specified	-.036	-.034	-.037	-.031	-.029
	(.045)	(.044)	(.044)	(.045)	(.045)
Other treatments:					
First generation		.079*	.077*	.072*	.073*
		(.041)	(.041)	(.041)	(.041)
Female		.056**	.060**	.058**	.058**
		(.025)	(.025)	(.025)	(.025)
Higher grade		-.039	-.034	-.034	-.033
		(.025)	(.025)	(.025)	(.025)
Performance signal		.029	.029	.033	.034
		(.025)	(.025)	(.025)	(.025)
Warmth signal		-.015	-.013	-.012	-.013
		(.025)	(.025)	(.025)	(.025)
Christian religion		-.019	-.022	-.022	-.022
		(.025)	(.025)	(.025)	(.025)
Nuts1 (ref. London)					
North East England			.055	.004	.001
			(.095)	(.095)	(.095)
North West England			-.120***	-.143***	-.143***
			(.045)	(.047)	(.047)
Yorkshire Humber			-.079	-.099*	-.1*
			(.056)	(.058)	(.058)
East Midlands			-.063	-.084	-.085
			(.057)	(.06)	(.06)
West Midlands			-.065	-.107*	-.107*
			(.057)	(.06)	(.06)
East of England			-.076*	-.104**	-.104**

			(.043)	(.045)	(.045)
South East England			-.032	-.059	-.058
			(.038)	(.041)	(.041)
South West England			-.118**	-.153***	-.153***
			(.046)	(.050)	(.050)
Wales			-.042	-.044	-.043
			(.134)	(.147)	(.147)
Scotland			-.294***	-.305***	-.302***
			(.083)	(.084)	(.085)
Average daily competition (ref. <1)					
1 to 2				-.033	-.033
				(.031)	(.031)
2 to 5				-.041	-.040
				(.045)	(.045)
5 to 10				-.057	-.056
				(.049)	(.049)
More than 10				-.140**	-.139**
				(.056)	(.056)
Time dummies (ref. 1st semester)					
2nd semester					-.011
					(.037)
3rd semester					-.013
					(.036)
Constant	.432***	.426***	.477***	.490***	.500***
	(.042)	(.051)	(.055)	(.058)	(.063)
N applicants	1111	1111	1111	1096	1096
R-squared	.060	.071	.083	.086	.086

Robust standard errors are in parentheses
**** p<.01, ** p<.05, * p<.1*

Source: GEMM data, own calculations.
Dependent variable: any interest from employers.
EU12 countries: France, Germany, Greece, Ireland, Italy, Netherlands, Spain.
Eastern EU countries: Bulgaria, Poland, Romania.

Table A4. Discrimination of European applicants (invitation to interview). Full table.

	M1	M2	M3	M4	M5
EU-country origin	-.017	-.057**	-.055**	-.056**	-.06**
	(.021)	(.024)	(.024)	(.024)	(.024)
Occupation (ref. cook)					
Payroll clerk	-.152***	-.148***	-.156***	-.136***	-.138***
	(.038)	(.038)	(.038)	(.043)	(.043)
Receptionist	-.141***	-.14***	-.16***	-.13***	-.134***
	(.042)	(.042)	(.041)	(.046)	(.046)
Sales representative	-.134***	-.134***	-.137***	-.128***	-.126***
	(.042)	(.042)	(.041)	(.043)	(.043)
Software developer	-.088**	-.089**	-.089**	-.086*	-.086*
	(.045)	(.045)	(.044)	(.045)	(.045)
Store assistant	-.085*	-.086*	-.088**	-.075	-.078*
	(.045)	(.045)	(.044)	(.046)	(.046)
Contract (ref. full time)					
Part time	-.063**	-.063**	-.06**	-.073***	-.074***
	(.028)	(.028)	(.028)	(.027)	(.027)
Flexible	-.020	-.018	-.012	-.005	-.008
	(.045)	(.045)	(.044)	(.044)	(.044)
Not specified	-.065**	-.064**	-.07**	-.069**	-.056*
	(.03)	(.03)	(.03)	(.03)	(.031)
Other treatments:					
First generation		.077**	.071**	.073**	.077**
		(.033)	(.033)	(.033)	(.033)
Female		.012	.016	.011	.012
		(.02)	(.02)	(.02)	(.02)
Higher grade		-.039*	-.032	-.03	-.027
		(.02)	(.02)	(.02)	(.02)
Performance signal		.017	.016	.018	.019
		(.02)	(.02)	(.02)	(.02)
Warmth signal		-.008	-.007	-.009	-.013
		(.02)	(.02)	(.02)	(.02)
Christian religion		-.014	-.015	-.014	-.015
		(.02)	(.02)	(.02)	(.02)
Nuts1 (ref. London)					
North East England			.083	.028	.015
			(.092)	(.092)	(.091)
North West England			-.134***	-.157***	-.159***
			(.035)	(.038)	(.037)
Yorkshire Humber			-.162***	-.183***	-.183***
			(.037)	(.039)	(.039)
East Midlands			-.074	-.097*	-.098*
			(.049)	(.052)	(.052)
West Midlands			-.095**	-.108**	-.109**
			(.046)	(.05)	(.049)
East of England			-.086**	-.106***	-.103***
			(.037)	(.039)	(.039)
South East England			-.099***	-.126***	-.125***
			(.031)	(.034)	(.034)
South West England			-.148***	-.182***	-.177***
			(.035)	(.038)	(.038)

Wales			-.203***	-.234***	-.220***
			(.037)	(.042)	(.042)
Scotland			-.212***	-.233***	-.217***
			(.051)	(.052)	(.053)
Average daily competition (ref. <1)					
1 to 2				-.007	-.004
				(.025)	(.025)
2 to 5				-.032	-.027
				(.035)	(.035)
5 to 10				-.004	.002
				(.045)	(.045)
More than 10				-.145***	-.133***
				(.043)	(.044)
Time dummies (ref. 1st semester)					
2nd semester					-.083***
					(.031)
3rd semester					-.068**
					(.031)
Constant	.253***	.270***	.351***	.370***	.428***
	(.037)	(.043)	(.047)	(.049)	(.054)
N applicants	1111	1111	1111	1096	1096
R-squared	.030	.039	.071	.078	.085

Robust standard errors are in parentheses
**** p<.01, ** p<.05, * p<.1*

Source: GEMM data, own calculations.
Dependent variable: invitation to interview.
EU countries: Bulgaria, France, Germany, Greece, Ireland, Italy, Netherlands, Poland, Romania, Spain.

Table A5. Discrimination of European applicants (invitation to interview): a comparison of EU12 and Eastern European migrants. Full table.

	M1	M2	M3	M4	M5
Origin (Ref. White British)					
EU12 origin	-.019	-.057**	-.057**	-.059**	-.064***
	(.023)	(.025)	(.025)	(.025)	(.024)
Eastern EU origin	-.012	-.058	-.049	-.047	-.049
	(.036)	(.04)	(.04)	(.04)	(.039)
Occupation (ref. cook)					
Payroll clerk	-.152***	-.148***	-.156***	-.136***	-.138***
	(.038)	(.038)	(.038)	(.043)	(.043)
Receptionist	-.141***	-.14***	-.159***	-.129***	-.134***
	(.042)	(.042)	(.041)	(.046)	(.046)
Sales representative	-.134***	-.134***	-.136***	-.128***	-.125***
	(.042)	(.042)	(.041)	(.043)	(.043)
Software developer	-.088**	-.089**	-.089**	-.086*	-.085*
	(.045)	(.045)	(.044)	(.045)	(.045)
Store assistant	-.085*	-.086*	-.088**	-.075	-.078*
	(.045)	(.045)	(.044)	(.046)	(.046)
Contract (ref. full time)					
Part time	-.064**	-.063**	-.06**	-.073***	-.074***
	(.028)	(.028)	(.028)	(.027)	(.027)
Flexible	-.02	-.018	-.012	-.005	-.008
	(.045)	(.045)	(.044)	(.044)	(.044)
Not specified	-.065**	-.064**	-.07**	-.069**	-.056*
	(.03)	(.03)	(.03)	(.03)	(.031)
Other treatments:					
First generation		.077**	.071**	.073**	.076**
		(.033)	(.034)	(.034)	(.033)
Female		.012	.016	.01	.011
		(.02)	(.02)	(.02)	(.02)
Higher grade		-.039*	-.032	-.03	-.027
		(.02)	(.02)	(.02)	(.02)
Performance signal		.017	.016	.018	.019
		(.02)	(.02)	(.02)	(.02)
Warmth signal		-.008	-.007	-.009	-.013
		(.02)	(.02)	(.02)	(.02)
Christian religion		-.014	-.015	-.014	-.015
		(.02)	(.02)	(.02)	(.02)
Nuts1 (ref. London)					
North East England			.083	.028	.016
			(.092)	(.092)	(.091)
North West England			-.134***	-.157***	-.158***
			(.035)	(.038)	(.037)
Yorkshire Humber			-.162***	-.184***	-.184***
			(.037)	(.039)	(.039)
East Midlands			-.074	-.097*	-.098*
			(.049)	(.052)	(.052)
West Midlands			-.095**	-.109**	-.11**
			(.046)	(.049)	(.049)
East of England			-.086**	-.106***	-.103***
			(.037)	(.039)	(.039)
South East England			-.099***	-.126***	-.124***

			(.031)	(.034)	(.034)
South West England			-.148***	-.182***	-.178***
			(.035)	(.038)	(.038)
Wales			-.204***	-.234***	-.22***
			(.037)	(.042)	(.042)
Scotland			-.213***	-.235***	-.22***
			(.051)	(.052)	(.053)
Average daily competition (ref. <1)					
1 to 2				-.008	-.005
				(.025)	(.025)
2 to 5				-.032	-.027
				(.035)	(.035)
5 to 10				-.004	.002
				(.045)	(.045)
More than 10				-.146***	-.134***
				(.044)	(.044)
Time dummies (ref. 1st semester)					
2nd semester					-.084***
					(.031)
3rd semester					-.067**
					(.031)
Constant	.253***	.27***	.351***	.37***	.428***
	(.037)	(.043)	(.047)	(.049)	(.054)
N applicants	1111	1111	1111	1096	1096
R-squared	.030	.039	.071	.078	.086

Robust standard errors are in parentheses
**** p<.01, ** p<.05, * p<.1*

Source: GEMM data, own calculations.
Dependent variable: invitation to interview.
EU12 countries: France, Germany, Greece, Ireland, Italy, Netherlands, Spain.
Eastern EU countries: Bulgaria, Poland, Romania.

Figure A1. Example of a CV and cover letter.

Marek Kowalski

Address, London
Mobile: [mobile number] | **Email:** [email address]

Personal statement

A qualified cook with over four years of experience, now looking for opportunities to further develop in the hospitality sector. A self-starter, able to work on own initiative and as part of a team.

Work Experience

July 2014 - present
Chef de Partie, *Hotel xxxx, London*

• Assisting the head chef in the day-to-day running of the kitchen
• Preparing and cooking meals
• Following recipes to meet restaurant's standards
• Strictly complying with food quality and temperature regulations
• Overseeing kitchen administration and stock management

September 2012 - June 2014
Commis Chef, *xxxx Hospital, London*

• Washed, peeled and trimmed food for cold buffets and side dishes
• Ensured that all food served was arranged properly and met quality standards
• Checked and ordered stock from suppliers, unloaded deliveries and organized the storeroom
• Checked the temperature of the fridge and followed safety and hygiene protocols

Education

2010-2012
College of xxxx London
Level 2 Diploma in Professional Cookery (City & Guilds)

2005-2010
xxxx High School, xxxx, London

Skills

Computer skills: Microsoft Office, Outlook
Training: Essential Food Hygiene certificate, COSHH training
Language skills: *Bilingual* English *and Polish* | French (basic)
Driving: Full, clean UK driving license; in possession of own car

Volunteer work

January 2014 - present
Volunteer at Youth Enrichment Project
Assisting with cooking and preparation of meals for various events like open days and local fairs.

References

Available on request

Dear Sir or Madam,

I would like to apply for the position of [*job title*] which I have seen advertised on [*online job platform*]. I have worked for four years as a cook in kitchens and dining facilities. I like my current job but I am seeking new challenges in a role that would allow me to reach my full potential. I would be happy to relocate.

As a chef de partie at [*name of current employer*] I prepare, cook and serve meals to the highest standards required. Over the years I have improved my cooking skills with a la carte menu and also gained experience in buffets and catering. I understand the importance of health and safety regulations and basic food hygiene procedures. I always make sure that food is stored and chilled properly and that the kitchen and the equipment are kept clean.

I am very flexible and I am happy to work outside of regular working hours or at weekends. I can work well on my own but I also enjoy being in a team.

I am a passionate cook both in my professional life and in my spare time. This is shown through my volunteer work at my *local* community centre, where I help prepare meals for various events like local fairs and open days.

Thank you for taking the time to consider my application. I am keen to discuss my experience and skills with you in more detail. Note that although I have a *Polish* background all my education and training has been in Britain *since the age of 6*.

I look forward to hearing from you.

Yours faithfully,
Marek Kowalski
